# The influence of sodium-glucose co-transporter-2 inhibitors on the risk of cancer therapy-related cardiac dysfunction: A meta-analysis

**DOI:** 10.17305/bb.2025.11847

**Published:** 2025-03-10

**Authors:** Zhitao Yan, Xiaona Xing, Jinmei Huang

**Affiliations:** 1 Department of Cardiology, Chongqing Jiangjin District Central Hospital/Chongqing University Affiliated Jiangjin Hospital, Chongqing, China; 2 Department of Cardiology, Guangyuan Central Hospital, Guangyuan, China; 3 Department of Clinical Nutrition, Chongqing Jiangjin District Central Hospital/Chongqing University Affiliated Jiangjin Hospital, Chongqing, China

**Keywords:** Sodium-glucose co-transporter-2 inhibitors, SGLT2, cancer therapy-related cardiac dysfunction, CTRCD, heart failure, HF, cancer, meta-analysis

## Abstract

Cancer therapy-related cardiac dysfunction (CTRCD) is a major concern for patients undergoing cardiotoxic cancer treatments. Sodium-glucose co-transporter-2 (SGLT2) inhibitors have shown cardioprotective effects in both diabetic and non-diabetic populations. However, their impact on CTRCD risk remains uncertain. This meta-analysis aimed to assess the association between SGLT2 inhibitor use and CTRCD in cancer patients receiving cardiotoxic treatments. A systematic search of PubMed, Embase, and Web of Science was conducted to identify relevant studies. Cohort studies comparing CTRCD incidence in cancer patients with and without SGLT2 inhibitor use were included. Risk ratios (RRs) were pooled using a random-effects model, and subgroup and meta-regression analyses were performed to explore potential effect modifiers. Ten cohort studies involving 34,847 cancer patients met the inclusion criteria. Overall, SGLT2 inhibitor use was associated with a significantly reduced risk of CTRCD (RR: 0.47, 95% confidence interval: 0.33–0.68, *P* < 0.001), though significant heterogeneity was observed (*I^2^* ═ 70%). Subgroup analysis indicated a stronger protective effect in patients receiving anthracyclines (RR: 0.26) compared to those undergoing other treatments (RR: 0.73, *P* for subgroup difference ═ 0.001). Additionally, the cardioprotective effect was more pronounced in cohorts with a lower proportion of men (<55%, RR: 0.27) compared to those with a higher proportion (≥55%, RR: 0.75, *P* < 0.001). Sensitivity analyses, conducted by excluding one study at a time, consistently supported these findings, reinforcing their robustness. In conclusion, SGLT2 inhibitor use is associated with a lower risk of CTRCD in cancer patients, particularly those receiving anthracyclines. These findings highlight the potential role of SGLT2 inhibitors in mitigating cardiotoxicity during cancer therapy.

## Introduction

Cancer therapy-related cardiac dysfunction (CTRCD) is a significant clinical concern, generally defined as a decline in left ventricular ejection fraction (LVEF) of more than 10% from baseline, often accompanied by symptoms of heart failure (HF) [1, 2]. CTRCD arises from the cardiotoxic effects of various anticancer treatments, affecting approximately 5%–25% of patients depending on the treatment regimen and individual risk factors [3, 4]. As the population of cancer survivors grows, addressing CTRCD is critical, as it contributes to long-term morbidity and reduced survival in this vulnerable group [5]. Given its substantial burden, identifying preventative strategies is essential to optimizing outcomes for cancer patients [6]. Several classes of anticancer therapies are known to induce CTRCD, with anthracyclines being the most extensively studied [7]. While anthracyclines are a cornerstone of treatment for hematologic malignancies and solid tumors, their dose-dependent cardiotoxicity is a well-documented limitation [7, 8]. Other therapies, such as gonadotropin-releasing hormone agonists (GnRH-A), widely used in hormone-sensitive cancers like breast and prostate cancer [9], and immune checkpoint inhibitors (ICIs), which have revolutionized cancer treatment by activating the immune system against tumors, have also been linked to cardiovascular complications [10, 11]. ICIs, in particular, are associated with immune-mediated myocarditis and other unpredictable forms of cardiotoxicity [12]. The cardiotoxicity of these therapies underscores the urgent need for protective strategies to allow continued use of effective cancer treatments without compromising cardiac health.

Sodium-glucose co-transporter 2 (SGLT2) inhibitors, initially developed as antihyperglycemic agents for type 2 diabetes, have gained attention for their broad cardioprotective and renoprotective benefits [13, 14]. Mechanistically, they enhance myocardial energetics, reduce inflammation and oxidative stress, and mitigate maladaptive cardiac remodeling [15, 16]. Clinical trials have demonstrated their efficacy in reducing HF hospitalizations and cardiovascular mortality across diverse populations, including those with and without diabetes [17, 18]. These pleiotropic effects make SGLT2 inhibitors a promising therapeutic option for preventing CTRCD, particularly in patients undergoing potentially cardiotoxic cancer treatments [19, 20]. While preclinical studies suggest they may counteract cardiotoxicity through antioxidative and antifibrotic pathways, clinical evidence remains limited but is evolving [21]. Previous meta-analyses evaluating SGLT2 inhibitors in CTRCD prevention included only three to four studies and reported inconclusive results regarding their effect on HF incidence in cancer patients [22–24]. These analyses were constrained by small sample sizes, heterogeneous study designs, and a lack of robust long-term outcome data [22–24]. However, several well-conducted studies have since been published, offering new insights into the potential benefits of SGLT2 inhibitors in this context [25–30]. Given the expanded evidence base, this meta-analysis aims to systematically assess the association between SGLT2 inhibitor use and CTRCD risk. By synthesizing updated data, this study seeks to clarify the utility of SGLT2 inhibitors in preventing CTRCD and to inform clinical practice regarding cardioprotective strategies for cancer patients.

## Materials and methods

The study followed PRISMA 2020 guidelines [31] and the Cochrane Handbook for Systematic Reviews and Meta-Analyses [32] in its design, data collection, statistical analysis, and result interpretation. The protocol for this systematic review and meta-analysis is registered in PROSPERO under the identifier CRD42024619366.

### Database search

To identify studies relevant to this meta-analysis, we conducted a comprehensive search of the PubMed, Embase, and Web of Science databases using an extensive set of search terms. These included: (1) “sodium glucose transporter 2 inhibitor” OR “sodium glucose transporter II inhibitor” OR “SGLT2 inhibitor” OR “SGLT-2 inhibitor” OR “SGLT2” OR “sodium glucose cotransporter 2 inhibitors,” as well as specific drug names, such as “canagliflozin,” “dapagliflozin,” “empagliflozin,” “ertugliflozin,” “tofogliflozin,” “bexagliflozin,” “henagliflozin,” “ipragliflozin,” “licogliflozin,” “luseogliflozin,” “remogliflozin,” “sergliflozin,” and “sotagliflozin”; (2) “chemotherapy” OR “anthracycline” OR “trastuzumab” OR “cancer” OR “tumor” OR “malignancy”; and (3) “cardiotoxicity” OR “cardiomyopathy” OR “heart failure” OR “cardiovascular” OR “cardiac dysfunction.” The search was restricted to studies involving human subjects and published in English. A detailed search strategy for each database is provided in Supplementary File 1. Additionally, we manually reviewed references from relevant original and review articles to identify further studies. The literature search covered publications from the inception of each database through November 14, 2024.

### Study inclusion

Inclusion criteria were established using the PICOS framework: P (Population): Adult cancer patients (aged 18 years or older) undergoing active cancer treatment, regardless of cancer type or treatment regimen, who are at risk of developing CTRCD. This includes patients receiving chemotherapy (e.g., anthracyclines), targeted therapy (e.g., trastuzumab), hormonal therapy (e.g., GnRH analogs), or ICIs. I (Intervention or Exposure): Use of SGLT2 inhibitors; C (Comparison): No use of SGLT2 inhibitors; O (Outcome): Incidence of CTRCD during follow-up, compared between cancer patients with and without SGLT2 inhibitor use. The definition and diagnosis of CTRCD were consistent with the criteria used in the original studies, typically defined as newly developed HF, cardiomyopathy, or a reduction in LVEF of more than 10% [3].

S (Study design): Randomized controlled trials (RCTs) or cohort studies, including both prospective and retrospective designs. Exclusion criteria included reviews, editorials, meta-analyses, preclinical studies, studies not limited to cancer patients, studies not including patients receiving cardiotoxic anticancer treatments, studies without a group receiving SGLT2 inhibitors, studies lacking a control group without SGLT2 inhibitor use, or studies that did not report CTRCD outcomes during follow-up. If multiple studies with overlapping populations were identified, the one with the largest sample size was selected for meta-analysis.

### Study quality assessment and data collection

Two authors independently conducted the literature search, study selection, quality assessment, and data extraction. Any disagreements were resolved through discussion with the corresponding author to reach a consensus. For RCTs, study quality was assessed using the Cochrane Risk of Bias tool, evaluating domains, such as random sequence generation, allocation concealment, blinding, and incomplete outcome data [32]. For cohort studies, quality was assessed using the Newcastle–Ottawa Scale (NOS) [33], which rates studies on a scale of 1–9 based on selection criteria, confounder control, and outcome assessment, with nine indicating the highest quality. Extracted data included study details (author, publication year, country, and design); patient demographics (cancer type, sample size, age, sex, and proportion with diabetes); concurrent cardiovascular medications that may prevent CTRCD, such as angiotensin-converting enzyme inhibitors (ACEIs), angiotensin II receptor blockers (ARBs), angiotensin receptor–neprilysin inhibitors (ARNIs) [34], and statins [35]; details of cardiotoxic anticancer treatments; follow-up durations; CTRCD definitions; the number of patients who developed CTRCD during follow-up; and variables adjusted or matched when evaluating the association between SGLT2 inhibitors and CTRCD risk.

**Figure 1.**
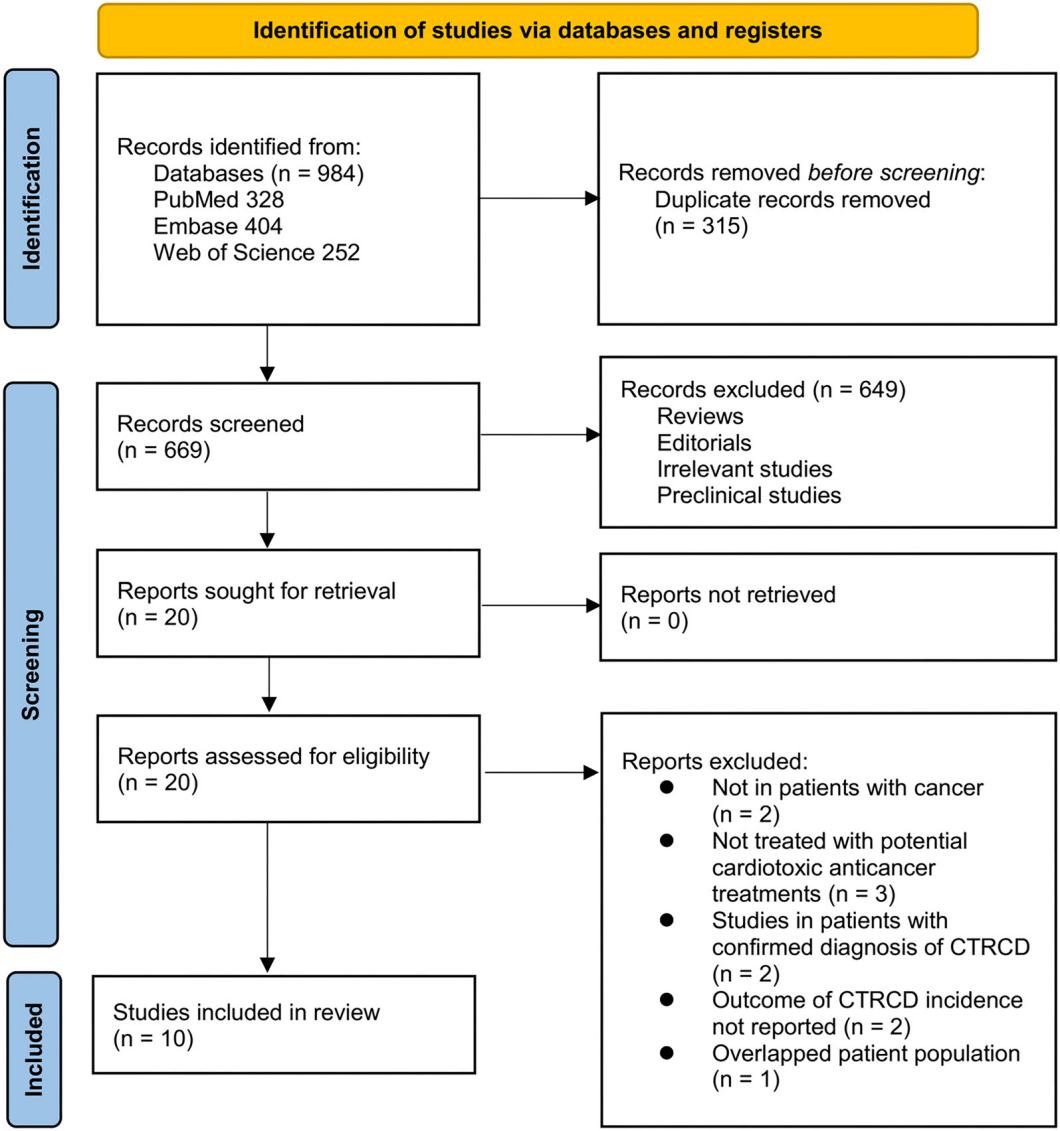
**Process of database search and study inclusion.** CTRCD: Cancer therapy-related cardiac dysfunction.

### Statistical analysis

The influence of SGLT2 inhibitors on the incidence of CTRCD in cancer patients, compared to those not using SGLT2 inhibitors, was assessed using the risk ratio (RR) and its corresponding 95% confidence interval (CI). RR values and their standard errors were derived from 95% CIs or *P* values and were subsequently logarithmically transformed to stabilize variance. Heterogeneity among studies was evaluated using the Cochrane *Q* test and *I*^2^ statistic, with an *I*^2^ > 50% indicating significant heterogeneity [36]. A random-effects model was applied to account for potential variations in patient characteristics and outcome definitions across studies. Sensitivity analysis was conducted by systematically excluding individual studies to assess result stability [32]. A univariate meta-regression analysis was performed to determine whether study characteristics—such as sample size, mean age, proportion of men, prevalence of diabetes, percentage of patients receiving ACEI/ARB/ARNI or statins, mean follow-up duration, and NOS scores—modified the association between SGLT2 inhibitors and CTRCD risk. Additionally, predefined subgroup analyses examined the impact of these variables on the meta-analysis outcomes, with medians of continuous variables used as cutoff values for subgroup definitions. Publication bias was assessed using funnel plots, visual inspection for asymmetry, and Egger’s regression test [37]. All statistical analyses were conducted using RevMan (Version 5.1; Cochrane Collaboration, Oxford, UK) and Stata (Version 17.0; Stata Corporation, College Station, TX, USA).

## Results

### Results of database search

Figure 1 provides a detailed overview of the study inclusion process. Initially, 984 potentially relevant records were identified across three databases. After removing 315 duplicate studies, the titles and abstracts of the remaining records were screened, leading to the exclusion of 649 studies that did not meet the meta-analysis objectives. The full texts of the remaining 20 studies were then assessed by two independent reviewers, resulting in the exclusion of 10 studies based on various criteria (as detailed in Figure 1). Ultimately, 10 cohort studies were deemed appropriate for inclusion in the quantitative analysis [25–30, 38–41].

**Table 1:** Study design and patient information of the included studies

**Study**	**Country**	**Design**	**Cancer type**	**Sample size**	**Mean age (years)**	**Men (%)**	**DM (%)**	**ACEI/ARB/ARNI (%)**	**Statins (%)**
Gongora, 2022	USA	RC	Lymphoma, breast, genitourinary, GI, sarcoma, leukemia, and other cancers	128	60	55.5	100	45.3	57
Chiang, 2023	Taiwan	RC	GI, genitourinary, thoracic, head and neck, breast, hematological, skin, and other cancers	1756	65	53	100	56	56
Abdel-Qadir, 2023	Canada	RC	Lymphoma, breast cancer, and others	933	71	37.8	100	72.6	76.4
Hwang, 2023	Korea	RC	Lymphoma, breast, genitourinary, and other cancers	8579	57.8	30.1	9.1	48.4	67.8
Fath, 2024	USA	RC	Hematological and lymphatic, breast, GI, genitourinary, skin, respiratory, and other cancers	1412	62.5	47	90	80.5	70.5
Daniele, 2024	Argentina	PC	Breast cancer with high risk of cardiac toxicity	76	68.6	0	84.2	98.5	72.5
Perelman, 2024	Israel	RC	NSCLC, RCC, HCC, melanoma, breast cancer and others	119	71	62	100	66	69
Koutroumpakis, 2024	USA	RC	Prostate cancer	4310	66.8	100	100	84.5	75.9
Bhatti, 2024	USA	RC	Breast cancer, lymphoma, hematological, GI, genitourinary, respiratory, and other cancers	17350	65.6	58.3	100	53.9	59.3
Chiang, 2024	Taiwan	RC	Colorectal cancer	184	68	66	100	48	47

DM: Diabetes mellitus; ACEI: Angiotensin-converting enzyme inhibitor; ARB: Angiotensin II receptor blocker; ARNI: Angiotensin receptor-neprilysin inhibitor; PR: Prospective cohort; RC: Retrospective cohort; GI: Gastrointestinal; NSCLC: Non-small cell lung cancer; RCC: Renal cell carcinoma; HCC: Hepatocellular carcinoma.

### Summary of the study characteristics

Tables 1 and 2 summarize the characteristics of the studies included in the meta-analysis. In total, one prospective cohort [27] and nine retrospective cohorts [25, 26, 28–30, 38–41] were analyzed, comprising 34,847 cancer patients. These studies, published between 2022 and 2024, were conducted in the United States, Taiwan, Canada, Korea, Argentina, and Israel. All included studies involved patients with various types of cancer, with ages ranging from 57.8 to 71.0 years. In seven studies [25–27, 30, 38–40], all patients had diabetes. The proportion of patients receiving ACEI/ARB/ARNI ranged from 45.3% to 98.5%, while statin use varied between 47.0% and 75.9%. Cardiotoxic anticancer treatments included anthracycline-based chemotherapy in five studies [27, 28, 38, 39, 41], while the remaining studies examined other treatments, such as GnRH-A [29], ICIs [30], and mixed chemotherapy/targeted therapy [25, 26, 40]. Overall, 13,478 patients (38.7%) used SGLT2 inhibitors. Follow-up durations ranged from 6.0 to 40.8 months (mean: 21.8 months), during which 2117 patients (6.1%) developed CTRCD. Potential confounding factors—such as age, sex, cancer type and stage, cardiovascular risk factors, and concurrent cardiovascular medications—were adjusted to varying degrees in the analysis of SGLT2 inhibitor use and CTRCD risk. The NOS scores for the included studies ranged from seven to eight, indicating good study quality (Table 3).

**Table 2:** Anticancer treatment and outcome of CTRCD of the included studies

**Study**	**Potential cardiotoxic therapy**	**No. of patients receiving SGLT2 inhibitors**	**Follow-up duration (months)**	**Definition of CTRCD**	**No. of patients developed CTRCD**	**Variables matched or adjusted**
Gongora, 2022	Anthracyclines	32	18	Newly developed cardiomyopathy (EF decrease >10% or <53%) or HF incidence	13	Age, sex, ethnicity, cancer type, cancer stage, CV risk factors, CV medications, and accumulating dose of doxorubicin
Chiang, 2023	Alkylating agents, antimetabolites, platinum, plant alkaloids, anthracyclines, TKIs, and ICIs	878	18.8	Newly diagnosed HF	24	Age, sex, cancer type, year of cancer diagnosis, institution treated, the presence of metastatic disease, underlying cardiac and non-cardiac comorbidities, use of CV medications and the type of cancer therapy
Abdel-Qadir, 2023	Anthracyclines	99	19	Newly diagnosed HF	93	Age, sex, year of chemotherapy, cancer type, CV risk factors, CV medications, and comorbidities
Hwang, 2023	Anthracyclines	779	40.8	HF hospitalization	62	Age, sex, indexed year, CV risk factors, use of CV medications, and cancer types
Fath, 2024	Anthracyclines	706	24	Newly diagnosed HF	74	Age, sex, ethnicity, cancer type, CV risk factors, comorbidities, and CV medications
Daniele, 2024	Anthracyclines	38	6	EF decrease >10% or <53%, or GLS decrease >15%	13	Age, sex, cancer stage and treatment, accumulating dose of doxorubicin, CV risk factors, and CV medications
Perelman, 2024	ICIs	24	28	HF exacerbation	7	Age, sex, cancer type, cancer stage, protocol therapy, CV risk factors, and CV medications
Koutroumpakis, 2024	Hormone therapy	2155	24	New onset HF (EF < 50%)	236	Age, sex, ethnicity, BMI, CV risk factors, comorbidities, anticancer treatment, and CV medications
Bhatti, 2024	Anthracyclines, antimetabolites, TKIs, proteasome Inhibitors, alkylating agents, monoclonal Ab, and aromatase inhibitor	8675	12	CTRCD (new onset cardiomyopathy or HF)	1594	Age, sex, ethnicity, CV risk factors, comorbidities, types of cancer, and CV medications
Chiang, 2024	Antimetabolites, platinum, and monoclonal Ab	92	32.8	New onset HF	1	Age, sex, cancer stage, PF, anticancer treatment, CV risk factors, comorbidities, and CV medications

SGLT2: Sodium-glucose co-transporter-2; CTRCD: Cancer treatment-related cardiac dysfunction; TKI: Tyrosine kinase inhibitor; ICI: Immune checkpoint inhibitor; Ab: Antibody; EF: Ejection fraction; HF: Heart failure; GLS: Global longitudinal strain; CV: Cardiovascular; PS: Performance status; BMI: Body mass index.

**Figure 2.**
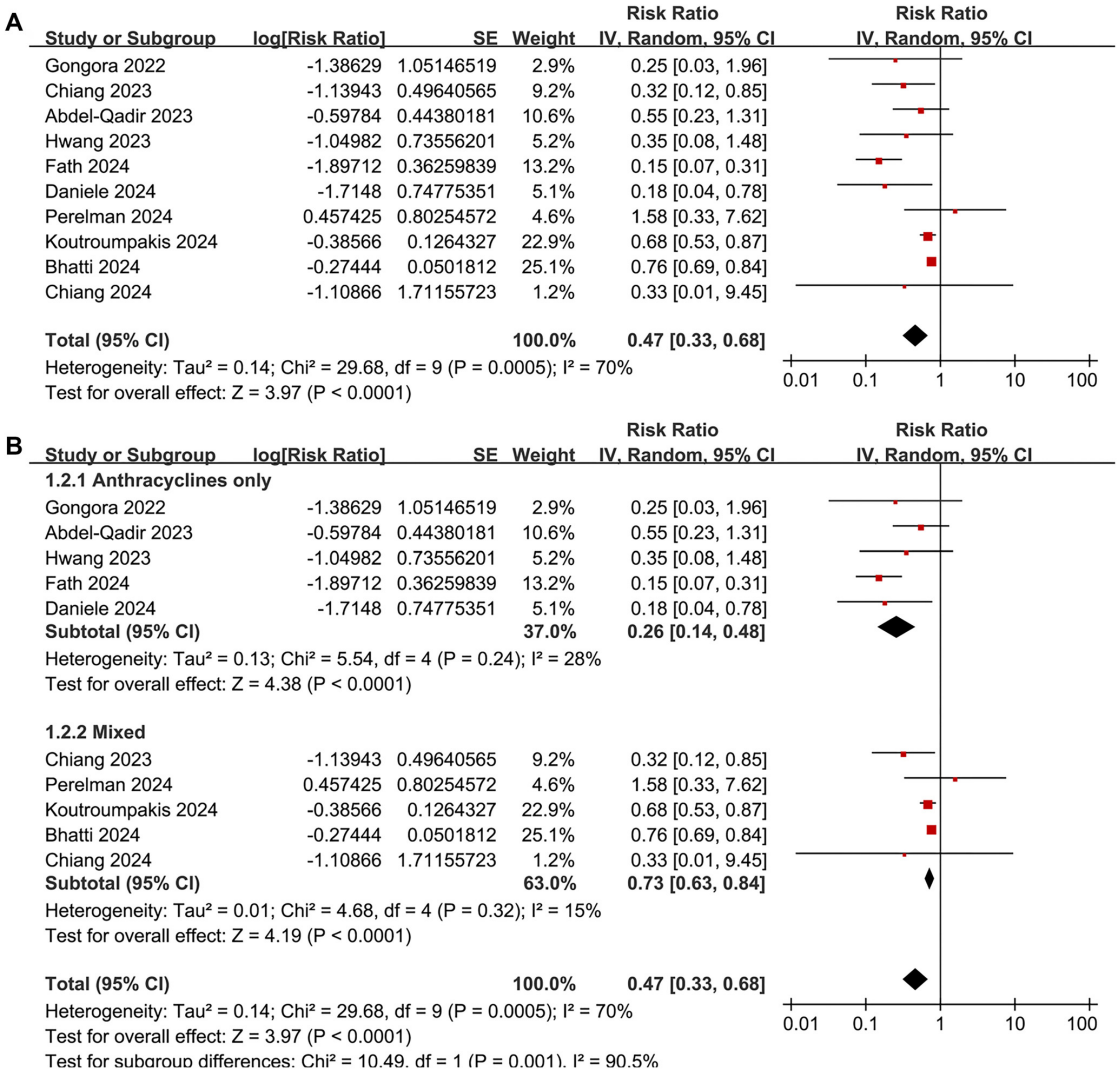
**Forest plots for the meta-analysis of the association between SGLT2 inhibitor use and the risk of CTRCD.** (A) Forest plots for the overall meta-analysis and (B) Subgroup analysis according to the potential cardiotoxic anticancer treatment used. CI: Confidence interval; SGLT2: Sodium-glucose co-transporter-2; CTRCD: Cancer therapy-related cardiac dysfunction.

### Meta-analysis results

**Figure 3.**
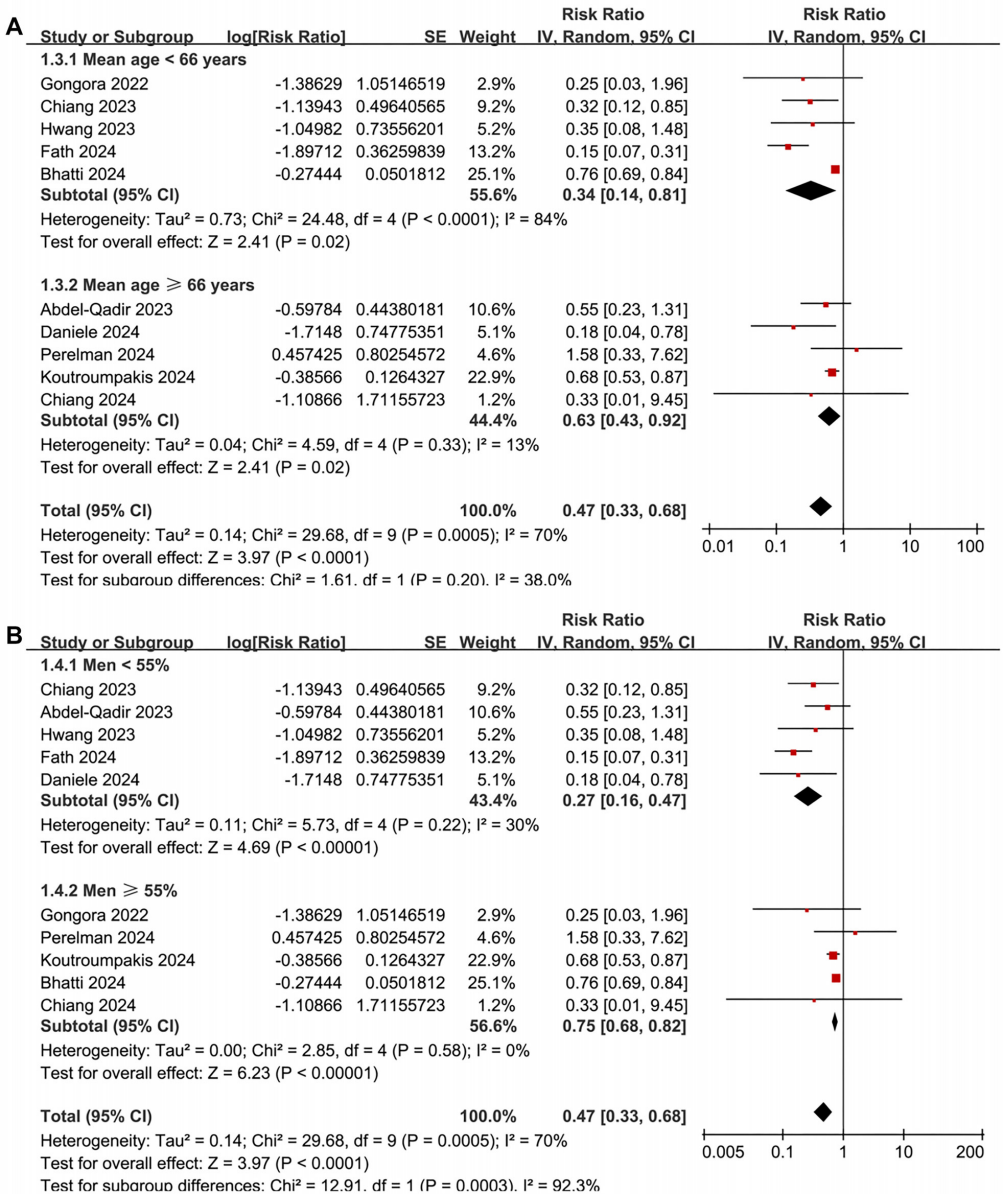
**Forest plots for the subgroup-analyses of the association between SGLT2 inhibitor use and the risk of CTRCD.** (A) Subgroup analysis according to the mean age of the patients and (B) Subgroup analysis according to the proportion of men in each study. CI: Confidence interval; SGLT2: Sodium-glucose co-transporter-2; CTRCD: Cancer therapy-related cardiac dysfunction.

**Figure 4.**
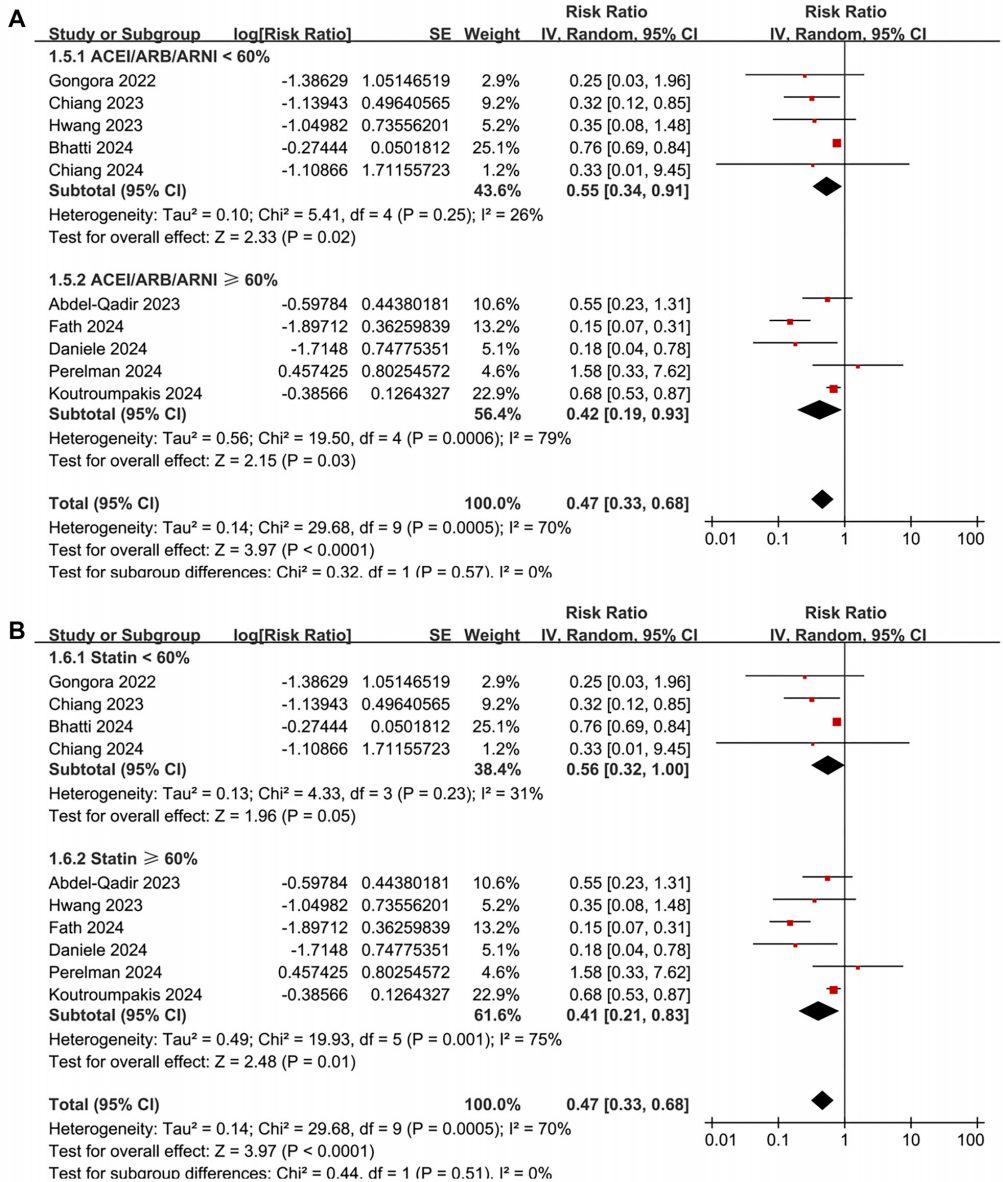
**Forest plots for the subgroup-analyses of the association between SGLT2 inhibitor use and the risk of CTRCD.** (A) Subgroup analysis according to the percentage of patients on ACEI/ARB/ARNI in each study and (B) Subgroup analysis according to the percentage of patients on statins in each study. CI: Confidence interval; SGLT2: Sodium-glucose co-transporter-2; CTRCD: Cancer therapy-related cardiac dysfunction; ACEI: Angiotensin-converting enzyme inhibitor; ARB: Angiotensin II receptor blocker; ARNI: Angiotensin receptor-neprilysin inhibitor.

**Figure 5.**
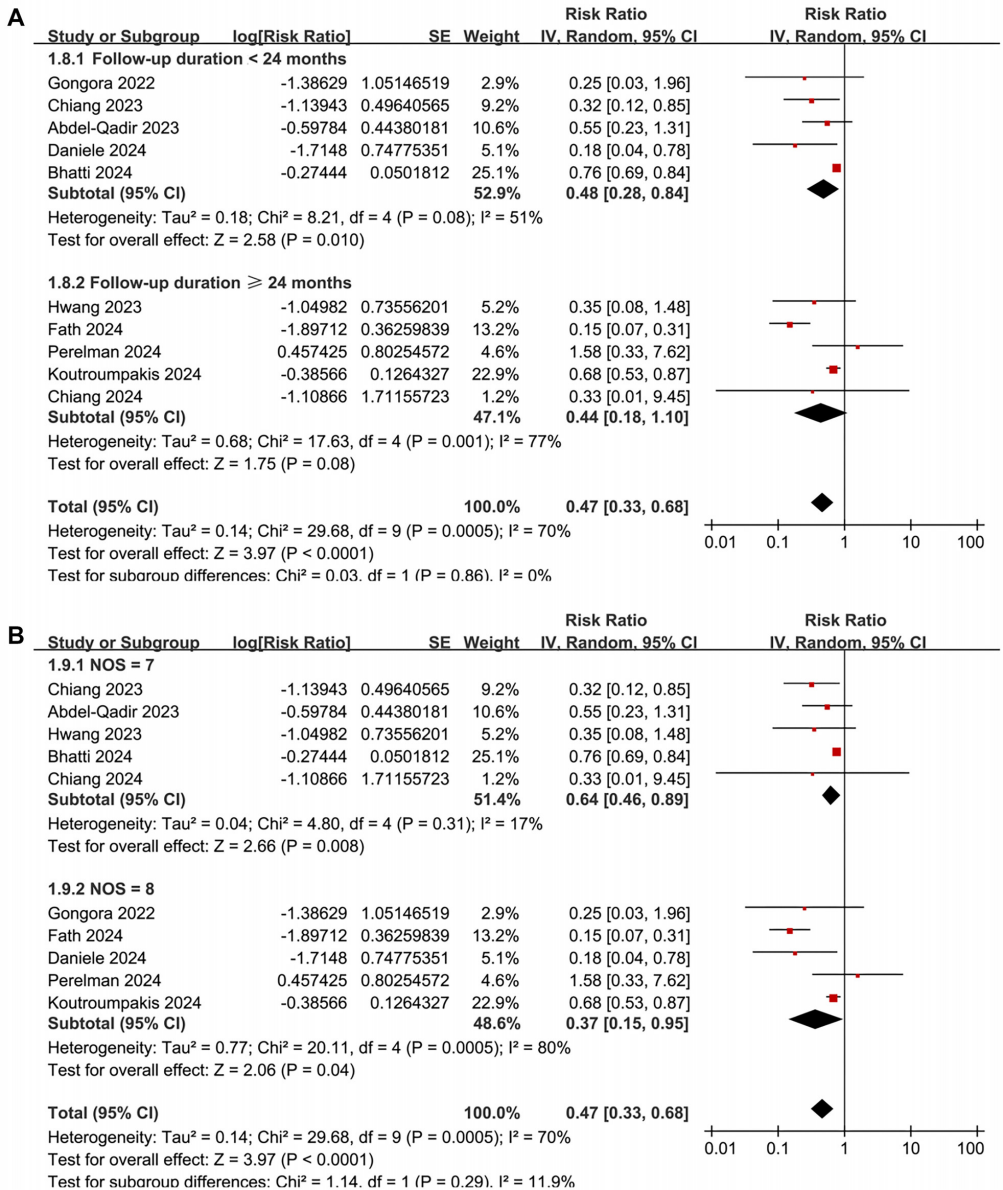
**Forest plots for the subgroup-analyses of the association between SGLT2 inhibitor use and the risk of CTRCD.** (A) Subgroup analysis according to the mean follow-up duration of each study and (B) Subgroup analysis according to the NOS scores of each study. CI: Confidence interval; SGLT2: Sodium-glucose co-transporter-2; CTRCD: Cancer therapy-related cardiac dysfunction; NOS: Newcastle-Ottawa Scale

A meta-analysis pooling results from 10 cohort studies [25–30, 38–41] found that SGLT2 inhibitor use was associated with a reduced risk of CTRCD in cancer patients (RR: 0.47, 95% CI: 0.33–0.68, *P* < 0.001; Figure 2A), though with significant heterogeneity (*I*^2^ ═ 70%). Sensitivity analysis, conducted by omitting one dataset at a time, did not significantly alter the results (RR: 0.39–0.66, *P* all < 0.05). Meta-regression analysis showed that study characteristics—including sample size, mean age, proportion of men, diabetes prevalence, percentage of patients on ACEI/ARB/ARNI or statins, follow-up duration, and NOS scores—did not significantly modify the association (all *P* > 0.05; Table 4). Subgroup analysis suggested a stronger association between SGLT2 inhibitor use and reduced CTRCD risk in patients receiving anthracyclines compared to those on mixed cardiotoxic anticancer treatments (RR: 0.26 vs 0.73, *P* for subgroup difference ═ 0.001; Figure 2B). Similar associations were observed in patients younger or older than 66 years (*P* ═ 0.20; Figure 3A). Notably, the protective effect was more pronounced in cohorts where men comprised <55% of the population compared to those with ≥55% (RR: 0.27 vs 0.75, *P* < 0.001; Figure 3B). Further subgroup analyses based on ACEI/ARB/ARNI use (*P* ═ 0.57; Figure 4A), statin use (*P* ═ 0.51; Figure 4B), follow-up duration (*P* ═ 0.86; Figure 5A), and NOS score (*P* ═ 0.29; Figure 5B) showed consistent results.

**Table 3:** Study quality evaluation via the Newcastle-Ottawa Scale

**Study**	**Representativeness of the exposed cohort**	**Selection of the non-exposed cohort**	**Ascertainment of exposure**	**Outcome not present at baseline**	**Control for age**	**Control for other confounding factors**	**Assessment of outcome**	**Enough long follow-up duration**	**Adequacy of follow-up of cohort**	**Total**
Gongora, 2022	0	1	1	1	1	1	1	1	1	8
Chiang, 2023	0	1	1	1	1	1	0	1	1	7
Abdel-Qadir, 2023	0	1	1	1	1	1	0	1	1	7
Hwang, 2023	0	1	1	1	1	1	0	1	1	7
Fath, 2024	0	1	1	1	1	1	1	1	1	8
Daniele, 2024	1	1	1	1	1	1	0	1	1	8
Perelman, 2024	1	1	1	1	1	1	0	1	1	8
Koutroumpakis, 2024	0	1	1	1	1	1	1	1	1	8
Bhatti, 2024	0	1	1	1	1	1	0	1	1	7
Chiang, 2024	0	1	1	1	1	1	0	1	1	7

**Table 4:** Results of univariate meta-regression analysis

**Variables**	**RR for the association between of SGLT2 inhibitor use and risk of CTRCD**			
	**Coefficient**	**95% CI**	***P* values**	**Adjusted *R*^2^**
Sample size	0.000045	−0.000038 to 0.00128	0.25	15.8%
Mean age (years)	0.094	−0.053 to 0.240	0.18	22.9%
Men (%)	0.013	−0.007 to 0.033	0.17	16.5%
Diabetes (%)	0.0074	−0.0169 to 0.0317	0.50	0.8%
ACEI/ARB/ARNI (%)	−0.010	−0.047 to 0.026	0.54	−9.1%
Statins (%)	0.0034	−0.0711 to 0.0778	0.92	−22.4%
Follow-up duration (months)	0.0022	−0.0688 to 0.0732	0.95	−18.3%
NOS	−0.29	−1.45 to 0.88	0.58	−11.1%

SGLT2: Sodium-glucose co-transporter-2; CTRCD: Cancer treatment-related cardiac dysfunction; RR: Risk ratio; CI: Confidence interval; ACEI: Angiotensin-converting enzyme inhibitor; ARB: Angiotensin II receptor blocker; ARNI: Angiotensin receptor-neprilysin inhibitor; NOS: Newcastle-Ottawa Scale.

### Publication bias

**Figure 6.**
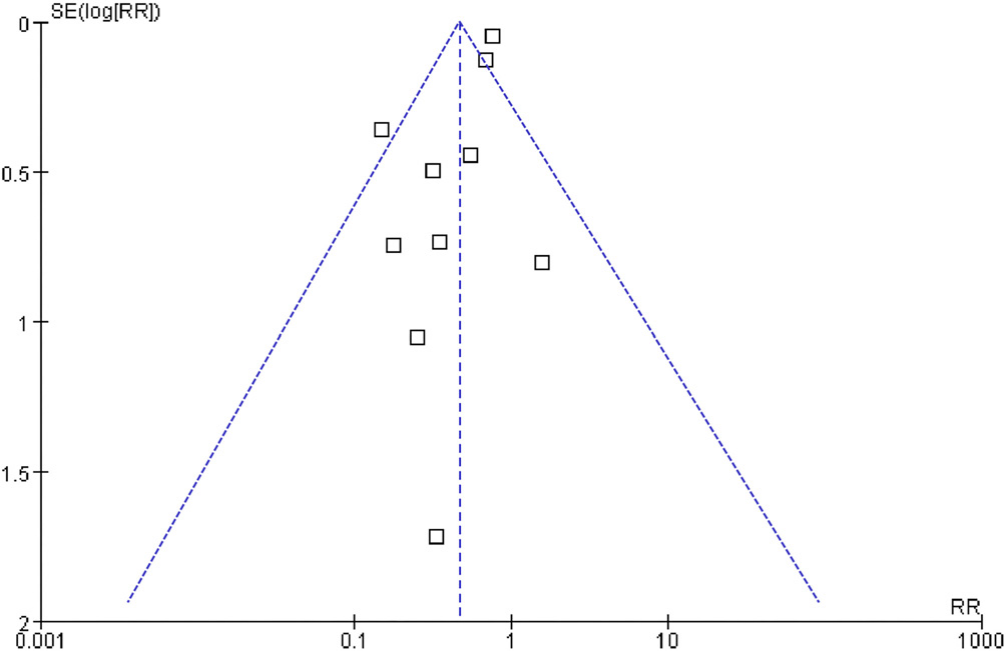
**Funnel plots for meta-analyses of the associations between SGLT2 inhibitor use and the risk of CTRCD**. CI: Confidence interval; SGLT2: Sodium-glucose co-transporter-2; CTRCD: Cancer therapy-related cardiac dysfunction; RR: Risk ratio.

A visual inspection of the funnel plots for the meta-analysis assessing the association between SGLT2 inhibitor use and the risk of CTRCD in patients with cancer showed symmetry, suggesting a low likelihood of publication bias (Figure 6). Additionally, Egger’s regression test indicated a low risk of publication bias (*P* ═ 0.18).

## Discussion

The findings of this meta-analysis demonstrate the potential of SGLT2 inhibitors to reduce the risk of CTRCD. By synthesizing data from diverse observational studies, we identified a significant association between SGLT2 inhibitor use and lower CTRCD incidence. Subgroup analyses revealed stronger efficacy in anthracycline-induced cardiotoxicity and suggested a potential trend toward greater protective effects in cohorts with a lower proportion of men. Notably, the observed benefits appeared independent of concurrent ACEI/ARB or statin use, reinforcing the unique cardioprotective mechanisms of SGLT2 inhibitors.

The cardioprotective effects of SGLT2 inhibitors in CTRCD are likely multifaceted [19]. A key mechanism is their ability to enhance cardiac energetics by shifting myocardial metabolism from glucose reliance to fatty acid oxidation and ketone body utilization, improving ATP production under stress conditions [42, 43]. This metabolic shift is particularly relevant in CTRCD, where mitochondrial dysfunction and energy deficits are common [44]. Moreover, SGLT2 inhibitors exert potent anti-inflammatory and antioxidative effects, which may help counteract the oxidative stress and inflammation induced by cancer therapies [45]. For example, anthracyclines—a major contributor to CTRCD—generate excessive reactive oxygen species (ROS), leading to lipid peroxidation, DNA damage, and mitochondrial dysfunction [46]. SGLT2 inhibitors may mitigate these effects by reducing ROS production and promoting mitochondrial biogenesis [47]. Additionally, their antifibrotic properties, mediated through inhibition of the sodium–hydrogen exchanger in cardiac myocytes, may help prevent maladaptive cardiac remodeling [48].

Our subgroup analysis suggested possible gender-specific responses to SGLT2 inhibitors, potentially influenced by hormonal differences. Estrogen has cardioprotective properties, including antioxidative and anti-inflammatory effects, which may synergize with the actions of SGLT2 inhibitors [49]. This could partially explain the stronger efficacy observed in cohorts with a higher proportion of women. However, the exact interplay between sex hormones and SGLT2 inhibitors in preventing CTRCD requires further investigation [50]. It is important to note that our subgroup analysis was based on the proportion of men at the study level rather than direct outcome comparisons between men and women. Moreover, meta-regression analysis did not show a significant correlation between the proportion of men and the effects of SGLT2 inhibitors in CTRCD prevention. Therefore, the possibility of gender-specific efficacy should be interpreted with caution [51]. While our findings suggest stronger benefits in cohorts with fewer men, this observation may be influenced by study-specific characteristics or other confounding factors [51]. Further research is needed to confirm these trends and clarify the underlying mechanisms. Subgroup and meta-regression analyses also provided additional insights into the context-specific efficacy of SGLT2 inhibitors. The stronger protective effect observed in anthracycline-related CTRCD aligns with the hypothesis that SGLT2 inhibitors target oxidative stress and mitochondrial dysfunction—key mechanisms of anthracycline-induced toxicity [52]. In contrast, their efficacy in hormone therapy- or ICI-induced CTRCD was less pronounced, suggesting that cardiotoxicity from these therapies, potentially driven by immune activation [53] or hormonal imbalances [54], may not be as effectively mitigated by SGLT2 inhibitors. These findings underscore the need for tailored cardioprotective approaches based on the specific cancer therapy used. Additionally, our analyses indicated that the protective effects of SGLT2 inhibitors were independent of concurrent ACEI/ARB [34] or statin [35] use, both of which are commonly prescribed to reduce cardiovascular risk. This suggests that SGLT2 inhibitors may provide unique and additive benefits beyond those of standard cardioprotective agents.

This meta-analysis has several strengths that enhance the reliability and applicability of its findings. A comprehensive literature search ensured a thorough assessment of available evidence. All included studies adjusted for key confounders using multivariate analyses, reducing bias. Multiple sensitivity analyses confirmed the robustness of the results, while subgroup and meta-regression analyses provided valuable insights into factors influencing the efficacy of SGLT2 inhibitors in preventing CTRCD. These strengths underscore the validity of the findings and their potential relevance to clinical practice. However, several limitations warrant consideration. The evidence synthesized primarily comes from observational studies, which, while informative, are prone to biases such as residual confounding and cannot establish causality. Large-scale RCTs are needed to validate these findings. Additionally, most included studies were retrospective, introducing potential selection and recall biases [55]. Future research should include prospective cohort studies and RCTs to confirm these results. Variability in SGLT2 inhibitor protocols—such as differences in drug type, dosage, timing, and duration—also limits the ability to recommend an optimal treatment regimen. Standardizing these protocols in future RCTs will be essential. Finally, the association between SGLT2 inhibitor use and reduced CTRCD risk may be confounded by unadjusted factors, such as variations in the dose and duration of cardiotoxic anticancer treatments. Future studies should explore the impact of these factors on SGLT2 inhibitor efficacy. From a clinical perspective, the findings of this meta-analysis have important implications. SGLT2 inhibitors, already established for their benefits in HF, chronic kidney disease, and type 2 diabetes, could be repurposed as a preventative strategy for CTRCD [56]. Their dual benefits in metabolic and cardiovascular health make them particularly attractive for cancer patients, who often have multiple comorbidities [57]. For instance, in anthracycline-treated patients at high risk of cardiac dysfunction, early initiation of SGLT2 inhibitors could potentially mitigate long-term cardiovascular complications. Their efficacy in preventing CTRCD may also extend to broader populations, including those undergoing novel cancer therapies such as ICIs or hormone therapy [58]. The potential for gender-specific efficacy of SGLT2 inhibitors warrants further investigation. If validated, this finding could inform personalized cardioprotective strategies, considering patient sex and hormonal status. Additionally, while SGLT2 inhibitors show independent benefits, their interactions with other cardioprotective agents—such as beta-blockers or novel anti-inflammatory drugs—remain unexplored [59]. Future high-quality RCTs should evaluate these combinations to determine the most effective strategies for CTRCD prevention.

## Conclusion

In conclusion, this meta-analysis provides preliminary evidence supporting the cardioprotective effects of SGLT2 inhibitors in reducing CTRCD risk, particularly in anthracycline-induced toxicity. By leveraging their unique mechanisms—such as improved cardiac energetics, antioxidative effects, and antifibrotic properties—SGLT2 inhibitors could be a valuable addition to the CTRCD prevention toolkit. However, the observational nature of the current evidence highlights the need for well-designed RCTs to validate these findings, optimize treatment protocols, and identify patient-specific factors influencing efficacy. With further research, integrating SGLT2 inhibitors into cardio-oncology practice could enhance the long-term cardiovascular health of cancer patients, addressing a critical unmet need in this population.

## Supplemental data

Detailed search syntax for each database

**PubMed**

(“sodium glucose transporter 2 inhibitor”[Mesh] OR “sodium glucose transporter ii inhibitor”[tiab] OR “SGLT 2 inhibitor”[tiab] OR “SGLT-2 inhibitor”[tiab] OR “SGLT2”[tiab] OR “sodium glucose cotransporter 2 inhibitors”[tiab] OR “canagliflozin”[tiab] OR “dapagliflozin”[tiab] OR “empagliflozin”[tiab] OR “ertugliflozin”[tiab] OR “tofogliflozin”[tiab] OR “bexagliflozin”[tiab] OR “henagliflozin”[tiab] OR “ipragliflozin”[tiab] OR “licogliflozin”[tiab] OR “luseogliflozin”[tiab] OR “remogliflozin”[tiab] OR “sergliflozin”[tiab] OR “sotagliflozin”[tiab]) AND (“chemotherapy”[Mesh] OR “anthracycline”[tiab] OR “trastuzumab”[tiab] OR “cancer”[tiab] OR “tumor”[tiab] OR “malignancy”[tiab]) AND (“cardiotoxicity”[Mesh] OR “cardiomyopathy”[tiab] OR “heart failure”[tiab] OR “cardiovascular”[tiab] OR “cardiac dysfunction”[tiab])

**Embase**

(“sodium glucose transporter 2 inhibitor”/exp OR “sodium glucose transporter ii inhibitor”:ti,ab OR “SGLT 2 inhibitor”:ti,ab OR “SGLT-2 inhibitor”:ti,ab OR “SGLT2”:ti,ab OR “sodium glucose cotransporter 2 inhibitors”:ti,ab OR “canagliflozin”:ti,ab OR “dapagliflozin”:ti,ab OR “empagliflozin”:ti,ab OR “ertugliflozin”:ti,ab OR “tofogliflozin”’:ti,ab OR “bexagliflozin”:ti,ab OR “henagliflozin”:ti,ab OR “ipragliflozin”:ti,ab OR “licogliflozin”:ti,ab OR ’luseogliflozin”:ti,ab OR “remogliflozin”:ti,ab OR “sergliflozin”:ti,ab OR “sotagliflozin”:ti,ab) AND (“chemotherapy”/exp OR “anthracycline”:ti,ab OR “trastuzumab”:ti,ab OR “cancer”:ti,ab OR “tumor”:ti,ab OR “malignancy”:ti,ab) AND (“cardiotoxicity”/exp OR “cardiomyopathy”:ti,ab OR “heart failure”:ti,ab OR “cardiovascular”:ti,ab OR “cardiac dysfunction”:ti,ab)

**Web of Science**

TS ═ (“sodium glucose transporter 2 inhibitor” OR “sodium glucose transporter ii inhibitor” OR “SGLT 2 inhibitor” OR “SGLT-2 inhibitor” OR “SGLT2” OR “sodium glucose cotransporter 2 inhibitors” OR “canagliflozin” OR “dapagliflozin” OR “empagliflozin” OR “ertugliflozin” OR “tofogliflozin” OR “bexagliflozin” OR “henagliflozin” OR “ipragliflozin” OR “licogliflozin” OR “luseogliflozin” OR “remogliflozin” OR “sergliflozin” OR “sotagliflozin”) AND TS ═ (“chemotherapy” OR “anthracycline” OR “trastuzumab” OR “cancer” OR “tumor” OR “malignancy”) AND TS ═ (“cardiotoxicity” OR “cardiomyopathy” OR “heart failure” OR “cardiovascular” OR “cardiac dysfunction”)

## Data Availability

The data that support the findings of this study are available from the corresponding author upon reasonable request.
